# Functional Physical Analysis and Quality of Life in the Preoperative and Early Postoperative Periods of Cardiac Surgery and 30 Days After Hospital Discharge

**DOI:** 10.21470/1678-9741-2022-0453

**Published:** 2024-05-13

**Authors:** Luana Gehm da Silva, Danieli Maria Magnaguagno, Mariana Motta Dias da Silva, Audrey Borghi-Silva, Eliane Roseli Winkelmann

**Affiliations:** 1 Graduate Program in Health Promotion, Universidade de Santa Cruz do Sul, Santa Cruz, Rio Grande do Sul, Brazil; 2 Undergraduate in Physiotherapy, Universidade Regional do Noroeste do Estado do Rio Grande do Sul, Ijuí, Rio Grande do Sul, Brazil; 3 Graduate Program in Statistics, Universidade Federal do Rio Grande do Sul, Porto Alegre, Rio Grande do Sul, Brazil; 4 Graduate Program in Physical Therapy (PPGFt), Universidade Federal de São Carlos, São Carlos, São Paulo, Brazil; 5 Graduate Program in Comprehensive Health Care (PPGAIS) (UNICRUZ, UNIJUI, URI), Universidade Regional do Noroeste do Estado do Rio Grande do Sul, Ijuí, Rio Grande do Sul, Brazil

**Keywords:** Thoracic Surgery, Respiratory Muscle Strength, Quality of Life, Cardiac Rehabilitation, Preoperative Period

## Abstract

**Introduction:**

The analysis of patients submitted to heart surgery at three assessment times has been insufficiently described in the literature.

**Objective:**

To analyze chest expansion, maximum inspiratory pressure (MIP), maximum expiratory pressure (MEP), distance traveled on the six-minute walk test (6MWT), and quality of life in the preoperative period, fourth postoperative day (4^th^ PO), and 30^th^ day after hospital discharge (30^th^-day HD) in individuals submitted to elective heart surgery.

**Methods:**

A descriptive, analytical, cross-sectional study was conducted with 15 individuals submitted to elective heart surgery between 2016 and 2020 who did not undergo any type of physiotherapeutic intervention in Phase II of cardiac rehabilitation. The outcome variables were difference in chest expansion (axillary, nipple, and xiphoid), MIP, MEP, distance on 6MWT, and quality of life. The assessment times were preoperative period, 4^th^ PO, and 30^th^-day HD.

**Results:**

Chest expansion diminished between the preoperative period and 4^th^ PO, followed by an increase at 30^th^-day HD. MIP, MEP, and distance traveled on the 6MWT diminished between the preoperative period and 4^th^ PO, with a return to preoperative values at 30^th^-day HD. General quality of life improved between the preoperative period and 4^th^ PO and 30^th^-day HD. An improvement was found in the social domain between the preoperative period and the 30^th^-day HD.

**Conclusion:**

Heart surgery causes immediate physical deficit, but physical functioning can be recovered 30 days after hospital discharge, resulting in an improvement in quality of life one month after surgery.

## INTRODUCTION

**Table:** 

Abbreviations, Acronyms & Symbols	
30^th^-day HD	= 30^th^ day after hospital discharge
4^th^ PO	= Fourth postoperative day
6MWT	= Six-minute walk test
ECC	= Extracorporeal circulation
ICU	= Intensive care unit
MEP	= Maximum expiratory pressure
MIP	= Maximum inspiratory pressure
SD	= Standard deviation

Cardiovascular diseases pose a considerable challenge for society, mainly due to the occurrence of comorbidities, constituting an important public health problem and one of the main reasons for hospitalization. In recent decades, cardiovascular diseases were responsible for 30% of all deaths, corresponding to 17 million people, according to data from the World Health Organization^[1]^.

The goals of heart surgery are to reduce symptoms, optimize heart function, and increase patient survival^[1]^. Although the aim is to prolong quality of life and optimize heart function, there are numerous negative impacts on functional capacity and lung function in the postoperative period^[2]^.

Heart surgery acutely leads to an intermittent decline in the oxygenation of tissues. Together with the period of hospitalization and the need for invasive mechanical ventilation, this can cause a reduction in lung compliance, generating an imbalance in the oxygenation of tissues and consequent negative impact on muscle function^[3]^.

In the study by Menezes et al.^[4]^, respiratory muscle weakness was a predictor of risk for the development of pulmonary complications. Heart surgery can magnify signs of respiratory muscle weakness in patients who have greater inspiratory muscle debility in the preoperative period, which is accentuated after the surgical procedure^[5]^. However, whether such problems persist 30 days after surgery remains to be investigated.

Therefore, the aim of the present study was to analyze chest expansion, maximum inspiratory pressure (MIP), maximum expiratory pressure (MEP), distance travelled on the six-minute walk test (6MWT), and quality of life in the preoperative period, fourth postoperative day (4 PO), and 30^th^ day after hospital discharge (30^th^-day HD) in individuals submitted to elective heart surgery.

## METHODS

### Type of Study

Descriptive, analytical, cross-sectional study conducted in the period from 2016 to 2020.

### Population

Patients submitted to heart surgery of myocardial revascularization or valve replacement.

### Setting

The present study was developed at the cardiology institute of a high-complexity hospital in the Northeast of Rio Grande do Sul, southern Brazil.

### Selection Criteria

Male and female heart patients aged ≥ 18 years were included. All participants were submitted to elective heart surgery between 2016 and 2020 at a medium-size hospital in the state of Rio Grande do Sul, Brazil, and performed the tests during the three assessments. The exclusion criteria were not completing all assessments, death during the data collection period, hospital stay longer than eight days, any type of complication, and having undergone any type of physiotherapeutic intervention in the post-discharge period (Phase II of cardiac rehabilitation).

### Data Collection

The patients submitted to heart surgery underwent assessments on three different occasions:

1) Preoperative period - on the day prior to surgery, data were collected from the patient records (identification, base disease, comorbidities, and risk factors), chest expansion and respiratory muscle strength were determined, the patients performed the 6MWT (done once), and a quality-of-life questionnaire was administered;

2) 4^th^ PO - with the patient in the ward, chest expansion and respiratory muscle strength were determined, the patients performed the 6MWT, and the quality-of-life questionnaire was administered;

3) 30^th^-day HD - the same measures were determined again.

#### Respiratory Muscle Strength

Determined by measuring MIP and MEP using the MVD-300 digital manometer (Microhard System, Globalmed, Porto Alegre, Rio Grande do Sul, Brazil). MIP and MEP were measured following the protocol described in previous studies^[6]^. Analysis involved absolute values and predicted values obtained from the equation proposed by Neder et al.^[7]^. MIP and MEP values > 70% of predicted were considered indicative of adequate respiratory muscle strength.

#### Chest Expansion

Chest circumference measurements were taken at the axillary, nipple, and xiphoid levels for the determination of chest expansion using inspiratory and expiratory measures^[8]^.

#### Quality of Life

It was assessed using the generic questionnaire proposed by the World Health Organization (the WHOQOL-bref)^[9]^ for the investigation of quality of life in adult populations. This instrument has 26 items, 24 of which are distributed among four domains: physical, psychological, social relations, and environment. There are also two general questions addressing the perception of quality of life and satisfaction with one’s health. Each item is scored from 1 to 5 points, with higher scores denoting a better quality of life.

#### Six-Minute Walk Test

This test is used to assess submaximal functional capacity by the longest distance an individual can walk in a fixed six-minute time interval. The 6MWT was performed along a 30-meter track following the recommendations of the America Thoracic Society^[10]^. Blood pressure and respiratory rate were measured at the beginning and end of the test. Heart rate, peripheral oxygen saturation (measured using a digital oximeter [ChoiceMMed Md300 Cn356 Vila Brasil] and attached to the patient’s finger during the entire test), and the Borg dyspnea scale were determined at the beginning, each minute, and end of the test. The calculation of the predicted distance for each individual was performed using the formula proposed by Enright and Sherrill et al.^[11]^.

### Data Analysis and Processing

Statistical analysis of the data was performed with the aid of the RStudio version 4.0.3. Qualitative variables were expressed as absolute and relative frequencies. Quantitative variables were expressed as mean and standard deviation. All variables related to chest expansion, respiratory muscle strength, and quality of life were tested for normality using the Shapiro-Wilk test. The Kruskal-Wallis test was used for the comparison of measures between assessment times (preoperative, 4^th^ PO, and 30^th^-day HD). The level of significance was set at 0.05.

### Ethical Aspects

This study was conducted in compliance with the regulatory norms for research involving human beings stipulated in Resolution 466/2012 of the Brazilian National Board of Health and the ethical precepts laid down in the Declaration of Helsinki. The study was developed using a databank from the institutional project approved by the Human Research Ethics Committee (protocol number: 39837020.4.0000.5350; approval number: 4.464.151/2020). All patients signed a statement of informed consent.

## RESULTS

**Fig. 1:**
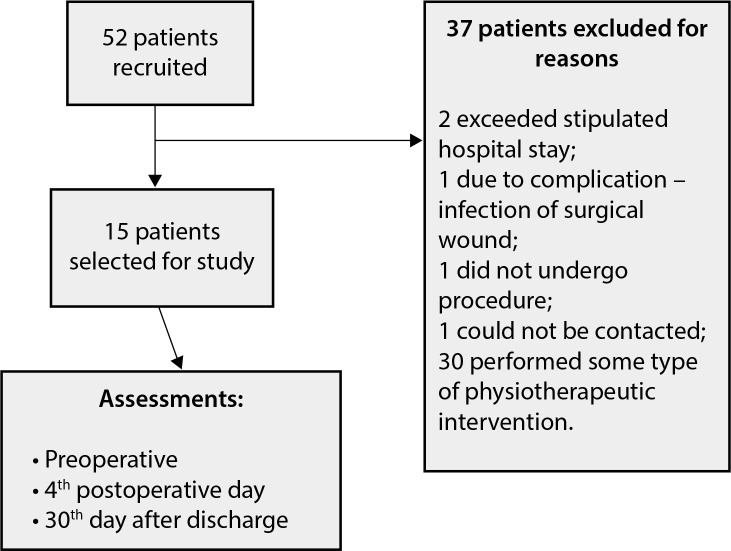
Flowchart of study.

Fifty-two patients were recruited, 37 of whom were excluded, and 15 participated in the study (Figure 1). Men predominated in the sample (66.67%). Mean age was 58 ± 8 years. Mean age and variability in the data were lower among women, whereas mean age was higher and with greater variability among men.

The most common risk factors were alcohol intake, cigarette smoking, and stress, each with 11 occurrences 11 (73,33%). More than half of the sample had risk factors. None of the patients were alcohol users at the time of the procedure.

In terms of surgery, myocardial revascularization accounted for the majority of cases (60%). The ejection fraction percentage was normal. Mean time of surgery, aortic cross-clamping time, and extracorporeal circulation are displayed in Table 1.

**Table 1:** Characterization of sample of individuals submitted to elective heart surgery.

	Women	Men	Total
Number of samples, n (%)	5 (33.33)	10 (66.67)	15 (100)
Age, mean ± SD	55.20 ± 6.46	59.10 ± 8.36	57.80 ± 7.78
Risk factors			
Current alcohol intake	0 (0.00)	0 (0.00)	0 (0.00)
Past alcohol intake	2 (13.33)	9 (60.00)	11 (73.33)
Smoker	4 (26.67)	7 (46.67)	11 (73.33)
Sedentarism	2 (13.33)	6 (40.00)	8 (53.33)
Stress	5 (33.33)	6 (40.00)	11 (73.33)
Surgery, n (%)			
Myocardial revascularization	2 (13.33)	7 (46.67)	9 (60.00)
Valve replacement	3 (20.00)	4 (26.67)	7 (53.33)
Intra-hospital variables, mean ± SD			
Time of surgery (minutes)	170.00 ± 58.88	227.0 ± 81.66	210.71 ± 78.30
Aortic cross-clamping time (minutes)	59.50 ± 24.75	70.00 ± 26.12	67.00 ± 25.25
Ejection fraction (%)	65.25 ± 14.43	61.70 ± 8.63	62.71 ± 10.12
ECC	75.50 ± 31.20	89.90 ± 28.00	85.79 ± 28.51
Time on mechanical ventilation (minutes)	697.50 ± 454.54	866.50 ± 793.52	818.21 ± 699.92
Time in ICU (days)	2.50 ± 1.00	2.11 ± 0.60	2.23 ± 0.73
Time in room (days)	3.25 ± 0.50	3.00 ± 0.50	3.08 ± 0.49
Total hospital stay (days)	5.75 ± 1.50	5.11 ± 0.60	5.31 ± 0.95

ECC=extracorporeal circulation; ICU=intensive care unit; SD=standard deviation

Mean hospital stay was 5.31 ± 0.95 days, with mean stay in intensive care of 2.23 ± 0.73 days and 3.08 ± 0.49 days in the room.

### Chest Expansion, Respiratory Muscle Strength, and 6MWT

The Kruskal-Wallis test for the comparison of means at the 5% level between the preoperative period, 4^th^ PO, and 30^th^-day HD revealed the following statistically significant differences: axillary, nipple, and xiphoid circumferences, MIP, % of predicted MIP, MEP, % of predicted MEP, % of predicted distance travelled on the 6MWT, general quality of life, and social domain of quality of life (Table 2).

**Table 2:** Comparison between preoperative period, 4^th^ PO, and 30^th^-day HD data of patients submitted to elective heart surgery.

Outcome variables, mean ± SD	Preoperative	4^th^- day PO	30^th^-day HD	*P*-value
Chest expansion				
Difference - axillary circumference	2.67 ± 1.59^ab^	1.87 ± 0.92^b^	4.00 ± 1.77^a^	0.0017^*^
Difference - nipple circumference	2.67 ± 1.63^ab^	1.60 ± 6.27^b^	2.93 ± 1.39^a^	0.0194^*^
Difference - xiphoid circumference	2.87 ± 1.77^ab^	1.60 ± 1.30^b^	3.73 ± 1.83^a^	0.0063^*^
Respiratory muscle strength				
MIP, cmH₂0	75.53 ± 39.33^a^	45.20 ± 26.89^b^	79.00 ± 38.84^a^	0.0151^*^
Predicted MIP, %	74.13 ± 39.44	43.13 ± 22.01	79.00 ± 40.06	0.0085^*^
MEP, cmH₂0	89.80 ± 38.59^a^	53.27 ± 22.20^b^	94.73 ± 44.70^a^	0.0122^*^
Predicted MEP, %	84.47 ± 35.14^a^	49.33 ± 16.67^b^	87.13 ± 36.47^a^	0.0028^*^
Distance on 6MWT	331.47 ± 85.79^a^	231.73 ± 75.34^b^	409.67 ± 98.24^a^	0.0000^*^
Predicted distance, %	67.20 ± 18.33^a^	47.60 ± 19.47^b^	83.73 ± 28.89^a^	0.0004^*^
Quality of life				
General	80.73 ± 18.99^b^	101.67 ± 14.97^a^	104.80 ± 10.83^a^	0.0004^*^
Physical domain	81.67 ± 12.44	84.07 ± 12.29	87.87 ± 17.19	0.3281
Psychological domain	88.00 ± 10.01	87.87 ± 9.55	92.13 ± 12.12	0.5665
Social domain	78.53 ± 21.52^b^	94.93 ± 19.67^ab^	106.53 ± 14.12^a^	0.0014^*^
Environmental domain	90.80 ± 10.58	96.20 ± 9.11	99.27± 11.86	0.105

30^th^-day HD=30^th^ day after hospital discharge; 4^th^-day PO=fourth postoperative day; 6MWT=six-minute walk test; MEP=maximum expiratory pressure; MIP=maximum inspiratory pressure; predicted MEP=percentage of the predicted maximum expiratory pressure achieved; predicted MIP=percentage of the predicted maximum inspiratory pressure achieved

a,b Equal letters mean there is no difference between the groups and different letters mean there is a difference between the groups

* Statistical significance when *P* ≤ 0.05

Chest expansion (axillary, nipple, and xiphoid) diminished between the preoperative period and the 4^th^ PO, followed by an increase on the 30^th^-day HD, with a statistically significant difference between the 4^th^ PO and the 30^th^-day HD.

Respiratory muscle strength (inspiratory and expiratory) assessed by MIP and MEP, the percentages reached of this strength, the distance travelled on the 6MWT, and the percentage of this distance reached were all significantly reduced between the preoperative period and the 4^th^ PO, with a statistically significant return to preoperative values on the 30^th^-day HD.

### Quality of Life

Quality of life had a different behavior. The data in Table 2 show an increase in the total score as well as all domain scores between the preoperative period and the 4^th^ PO and further increases on the 30^th^-day HD. However, statistically significant differences were found in general quality of life between the preoperative period and the 4^th^ PO as well as between the preoperative period and the 30^th^-day HD. A statistically significant difference was also found for the social domain between the preoperative period and the 30^th^-day HD.

## DISCUSSION

**Fig. 2:**
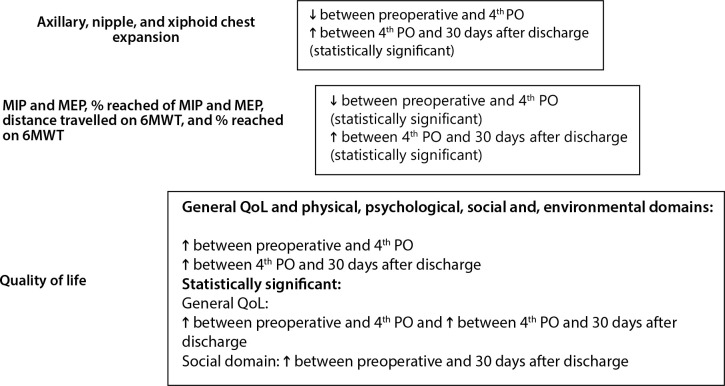
Summary of results. 6MWT=six-minute walk test; MEP=maximum expiratory pressure; MIP=maximum inspiratory pressure; 4^th^ PO=fourth postoperative day; QoL=quality of life.

This study showed the results of physical functional assessments of heart surgery patients on three different occasions (Figure 2), and these findings are important to clinical practice.

### Chest Expansion

Chest expansion (axillary, nipple, and xiphoid) diminished from the preoperative period to the postoperative period (time of discharge from hospital), with an increase between discharge and 30 days after surgery. Similar results are reported in the study by Pimenta et al.^[12]^, who found changes in lung compliance, with a reduction in circumference in the axillary and umbilical regions, which may occur due to postoperative pain, limiting the mobility of the rib cage and abdomen. Postoperative pain and fear associated with changes in pulmonary mechanics resulting from the surgical procedure hamper deep inspiration, with restrictions in respiratory movement.

### Respiratory Muscle Strength

Respiratory muscle strength reduced between the preoperative period and the 4^th^ PO, with a return to preoperative values on the 30^th^-day HD. In the study by Menezes et al.^[4]^, the authors describe a reduction in respiratory and peripheral muscle strength associated with heart surgery, which was directly linked to the pain of the surgical procedure. Nascimento et al.^[13]^ concluded that the reduction in respiratory muscle strength in the postoperative period is not completely reversed after heart surgery, which suggests the need for respiratory muscle training after the procedure.

Other studies report similar findings to the present results^[14-17]^. Urell et al.^[14]^ found that patients in the preoperative period had respiratory muscle strength within the predicted range, whereas an 11% reduction of the predicted value was found on the fifth postoperative day, and strength was recovered to predicted values two months after the surgical procedure. Carneiro et al.^[16]^ found a reduction in both MIP and MEP on the third postoperative day compared to the preoperative period, but reported a significant increase in MEP between the third and fifth postoperative day (*P*<0.05).

### Six-Minute Walk Test

Besides the measure of respiratory muscle strength, the determination of functional capacity employing the 6MWT is useful for a good physiotherapeutic assessment of patients submitted to heart surgery. According to Oliveira et al.^[18]^, patients with greater walking capacity in the postoperative period have a shorter hospital stay and the distance on the 6MWT is the best way to demonstrate the functional capacity of these individuals. Nery et al.^[19]^ found that the functional capacity of patients submitted to myocardial revascularization surgery improved substantially 30 days after the procedure - even surpassing preoperative values, which is similar to the present results.

Some findings on the 6MWT reveal interesting relationships. Gonçalves et al.^[20]^ found that patients in the preoperative period of heart surgery had a positive correlation between the 6MWT with respiratory muscle strength. Ramalho et al.^[21]^ assessed patients with heart failure and found that those who were unable to reach 350 m on the 6MWT were at greater risk of death within 10 years.

### Quality of Life

In a previous study by our research group^[5]^, we found a reduction in all variables analyzed in the physical functional assessment, including MIP, MEP, and chest expansion, between the preoperative and postoperative periods. However, quality of life values improved in the four domains (physical, psychological, social relations, and environmental) as well as the two general questions.

In the present study, quality of life had a different behavior compared to the functional analysis, as improvements were found in the general quality-of-life score as well as the domains scores between the preoperative period and discharge, and the scores continued to increase at 30 days after surgery, although statistically significant differences were only found for general quality of life and the social domain. According to Lisboa et al.^[22]^, heart surgery exerts a positive impact on the quality of life of patients in all domains, especially three months after the procedure. Moraes et al.^[23]^ found an increase in general quality of life and the physical domain six months after myocardial revascularization surgery, which translates to an increase in the capacity to perform activities that were not possible in the preoperative period. The improvement in these domains was greater in individuals who practiced physical activity regularly after the procedure.

In the study by Nogueira et al.^[24]^, quality of life was assessed using the same instrument used in this investigation, and improvements were found in the physical and mental components after the surgical procedure. However, differences between the sexes were found at the postoperative assessment, as women required a longer stay in intensive care, greater postoperative care, longer time on mechanical respiration, and a longer hospital stay as well as exhibiting a reduction in cognitive capacity. Nonetheless, both sexes demonstrated improvements in quality of life after surgery.

### Limitations

The present study shows the physical functional conditions and quality of life of heart surgery patients in the pre and postoperative periods and can therefore assist in improving clinical conduct. However, the following limitations should be considered: 1) the non-inclusion of all patients submitted to heart surgery during the data collection period, and 2) incompatibility of times to perform the tests on all patients. Thus, further studies on this issue with a larger number of participants are needed.

## CONCLUSION

Heart surgery causes immediate physical functional deficit, but physical functioning can be recovered 30 days after discharge from hospital, with a positive impact on quality of life one month after surgery. However, the study shows the need for a physical rehabilitation protocol to maintain and improve these results. We also know that if living standards are not improved, heart disease could return, and the patient will require another procedure.
